# Endoscopic Transverse Gastrocsoleus Recession in Children With Cerebral Palsy

**DOI:** 10.3389/fped.2020.00112

**Published:** 2020-03-24

**Authors:** Dae-Wook Kim, Hyun Woo Kim, Ji-Yeon Yoon, Isaac Rhee, Min-Kyung Oh, Kun-Bo Park

**Affiliations:** ^1^Department of Orthopaedic Surgery, Haeundae Paik Hospital, Inje University College of Medicine, Busan, South Korea; ^2^Division of Pediatric Orthopaedic Surgery, Severance Children's Hospital, Yonsei University College of Medicine, Seoul, South Korea; ^3^Medical Course, University of Melbourne, Melbourne Medical School, Melbourne, VIC, Australia; ^4^Clinical Trial Center, Busan Paik Hospital, Inje University College of Medicine, Busan, South Korea

**Keywords:** gastrocsoleus, vulpius lengthening, endoscopic surgery, spastic diplegia, equinus

## Abstract

**Aim:** The aim of this study was to evaluate the surgical outcome, in terms of gait improvement, of endoscopic transverse Vulpius gastrocsoleus recession in children with cerebral palsy compared to the traditional open surgery.

**Methods:** Twenty-seven children with cerebral palsy who had undergone endoscopic transverse Vulpius gastrocsoleus recession were reviewed. For the comparison of gait improvement, independent ambulatory spastic diplegic patients who had undergone only endoscopic transverse Vulpius gastrocsoleus recession on both legs were selected. Seven (14 legs) children were included and the median age was 7 years (6–9 years). Seven age-matched patients with the same inclusion/exclusion criteria who underwent open surgery were selected as the control group. Physical examination and gait parameters were evaluated and compared between groups, including the gait deviation index (GDI), and gait profile score (GPS).

**Results:** There was no significant complication in twenty-seven children after endoscopic transverse Vulpius gastrocsoleus recession. However, one patient required a revision open surgery at postoperative 1 year 9 months due to the recurrence of equinus and the incomplete division of the midline raphe which was noted during surgery. When comparing gait improvements, there were no differences between the endoscopic and open surgery groups in ankle dorsiflexion angle, ankle kinetics, GDI, and GPS. The postoperative peak ankle dorsiflexion during stance phase was slightly higher in the open group.

**Conclusion:** This is the first study that evaluates gait improvement exclusively for children with spastic diplegia after endoscopic transverse Vulpius gastrocsoleus recession. The gait improvements after endoscopic surgery were comparable to the open surgery, however, the possibility of reduced improvement in ankle kinematics should be considered.

## Introduction

For the correction of ankle equinus, there are several surgical methods, such as lengthening of the Achilles tendon, gastrocsoleus recession, and intramuscular lengthening of gastrocnemius or soleus. Gastrocsoleus recession results in greater muscle lengthening when compared with intramuscular lengthening and is also more stable than lengthening of the Achilles tendon in terms of preventing overcorrection (1). Recently, surgical outcome of endoscopic gastrocnemius recession has been reported as safe with minimal incision scarring (2–6).

A better cosmesis with a reduction in scar size may be one of the benefits of endoscopic surgery for children with cerebral palsy and their parents, as the traditional open surgery is associated with multiple incisions or hypertrophied scars (7). Grady et al. reported an acceptable outcome of endoscopic gastrocsoleus recession in children, but all the patients were neurologically healthy (3). An optimal gastrocsoleus recession is critical because of the risk of crouch gait from overcorrection, or the possibility of reoperation after the recurrence of equinus because of under correction in patients with cerebral palsy (8–11).

Poul et al. reported the improvement of ankle dorsiflexion angle after endoscopic gastrocsoleus recession in patients with cerebral palsy (2). However, this was determined by physical examination only and there was no description about gait improvement. And, there have been no definitive descriptions in previous studies about the diagnosis, such as spastic diplegia or spastic hemiplegia, and the surgical method used in the endoscopic gastrocsoleus recession, such as whether the Baumann, Strayer, Vulpius, or Baker method was utilized (8, 9, 11–13). Furthermore, patients with foot and ankle deformity should be excluded for the evaluation of surgical outcome after gastrocsoleus recession, because those may act as a selection bias.

The transverse Vulpius gastrocsoleus recession is an effective procedure for the correction of equinus gait in children with cerebral palsy and associated fixed gastrocsoleus contracture (12). Endoscopic transverse Vulpius gastrocsoleus recession was performed for patients with cerebral palsy who desired a small incision and minimal scarring in our institute. The purpose of this study was (1) to review the surgical outcome of endoscopic transverse Vulpius gastrocsoleus recession in children with cerebral palsy and (2) to evaluate and compare the gait improvement with open surgery in independent ambulatory spastic diplegic patients without foot and ankle bony deformities.

## Materials and Methods

### Subjects

This clinical study was approved by the Institutional Review Board of the research hospital (IRB No. 2016-10-012) and conformed to the requirements of the Declaration of Helsinki. All study participants were children whose parents or guardians provided informed consent for participation in the study.

Between 2012 and 2017, patients who had undergone endoscopic transverse Vulpius gastrocsoleus recession were reviewed and the first 10 patients were not included considering the surgeon's learning curve. Twenty-seven pediatric patients with cerebral palsy had undergone endoscopic transverse Vulpius gastrocsoleus recession. According to the gross motor function classification system (GMFCS) (14), 18 were classification II, 7 were III, and 2 patients were IV. Two patients were diagnosed with spastic hemiplegia and the others were spastic diplegia. To compare the gait improvement after endoscopic transverse Vulpius gastrocsoleus recession, independent ambulators were selected. The inclusion criteria were spastic diplegia, GMFCS II, patients who exclusively underwent simultaneous endoscopic transverse Vulpius surgery only for both legs with follow-up of more than 2 years. The exclusion criteria were patients with previous surgeries before the index operation, Botox injection within 6 months of the surgery, additional foot or lower extremity surgery, and additional orthopedic surgery during the follow-up period. Finally, seven patients (14 legs, 6–10 years) were included. The control group consisted of patients who had undergone open transverse Vulpius gastrocsoleus recession and met the same inclusion and exclusion criteria. Age-matched seven patients (14 legs) were selected in our database.

### Surgical Procedure

**Figure 1:**
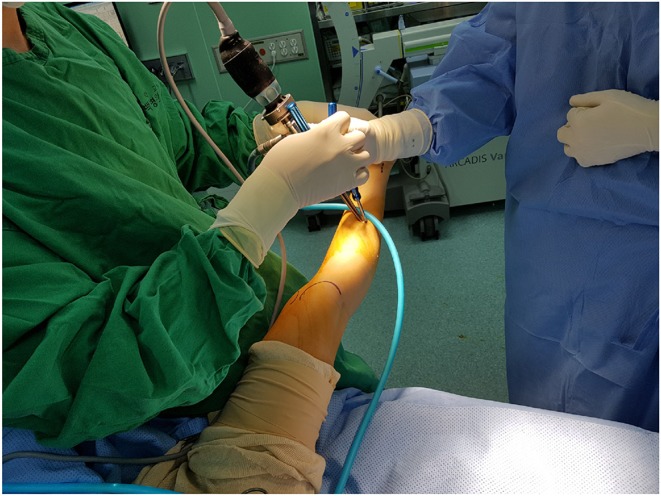
Endoscopic Vulpius surgery. The femoral condyle and Achilles tendon were identified and the skin incision at Zone 2 was marked. The carpal tunnel blade was inserted with an arthroscopy.

**Figure 2:**
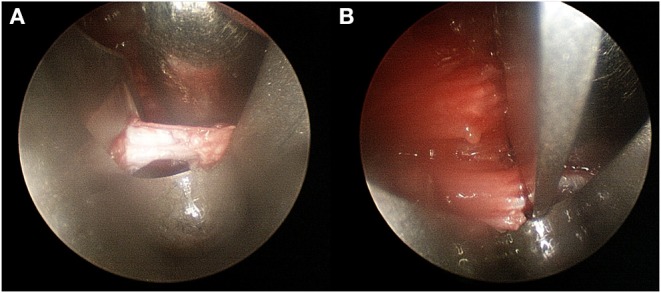
Intraoperative endoscopic view. **(A)** After transverse fascia incision, the blade was located. **(B)** At the end of fascia, complete fascia release, and muscle fiber were confirmed.

All operations were performed by a single surgeon (XXX). The indication of the transverse Vulpius gastrocsoleus recession were <10 degrees improvement of ankle dorsiflexion with increase of knee flexion or limited ankle dorsiflexion below 5 degrees with knee flexion. Open transverse Vulpius surgery was performed at anatomical zone 2 as previously described in the literature (8, 9, 12). The endoscopic transverse Vulpius surgery was performed first before the other single-event multilevel surgery (SEMLS) procedures, using an endoscopic carpal tunnel guided release system (GRS) (Arthrex, Inc., Naples, Florida, USA). Endoscopic transverse Vulpius surgery was performed at anatomical zone 2 as previously described for in the open procedure (3, 5, 6, 12, 13). Through a small longitudinal medial incision at zone 2, the deep fascia was divided, and a synovial elevator was used to protect the sural nerve and the short saphenous vein (Figure 1). Following a direct 5 mm transverse fascia incision, a 3.5 mm arthroscope was used for an endoscopic approach and carpal tunnel blade proceeded to transversely divide the conjoined tendon (Figure 2). If the ankle dorsiflexion was <10° of the knee extension after the release of the conjoined tendon, the midline raphé was divided under arthroscopic view. Postoperatively, if the patient did not undergo bony surgery, such as calcaneus, tibia or femur osteotomy as part of SEMLS, immediate weight bearing with a short-leg cast was used, if tolerated. The cast was changed to a solid ankle foot orthosis (AFO) at postoperative 4 weeks. When the patient could raise the heels and stand on one leg, the solid AFO was changed to a hinged AFO. At postoperative 12 months, the hinged AFO was removed. Final follow-up gait analysis was recommended after a postoperative period of 24 months.

### Evaluation of the Surgical Outcome in Independent Ambulatory Patients With Spastic Diplegia

Electronic medical records were reviewed to evaluate any complication related to the endoscopic surgery. Gait analysis was performed using the VICON 370 Motion Analysis System (Oxford Metrics, Oxford, UK) with six infra-red cameras and force platforms (Advanced Mechanical Technology, Watertown, MA, USA). Reflective markers were placed, by the same experienced examiner, over specified anatomical locations of the pelvis and lower extremities. All study subjects were asked to walk barefoot at a comfortable speed along a walkway that was 15 m in length, with markers in place along the walkway. Data from at least ten gait trials on this 15 m walkway were collected, from which, one representative cycle was chosen for analysis. Force-plates under the path recorded ground reaction forces during walking, and joint moments were expressed as internal moments to counter the ground reaction force. Muscle-tendon length was calculated using the Polygon software package (version 3.0, Oxford Metrics, Oxford, UK) using kinematic data. The standard generic model was scaled to the individual subject size by using three-dimensional kinematic data from pelvic anatomical landmarks. Muscle-tendon length was expressed as percentage ratio based on muscle-tendon length during gait vs. muscle-tendon length in the resting state (15). The average anatomical muscle-tendon lengths of the medial gastrocnemius and the lateral gastrocnemius muscles were measured during the single-limb support phase.

Preoperative and final follow-up values were used for comparison. The ankle dorsiflexion angle with full knee extension and with 90° knee flexion were compared. For gait parameters, maximum ankle dorsiflexion angle and maximum ankle plantarflexion moment were selected, and the maximum muscle-tendon length of the medial and lateral gastrocnemius during the stance phase were calculated and compared. As described in the literature the gait deviation index (GDI) and the gait profile score (GPS) were calculated and compared (16, 17). The operation time and scar length at the final follow-up were evaluated.

### Statistical Analysis

Statistical analysis was performed using the SPSS statistics software package, version 23 (IBM Corp, Armonk, NY, USA). To minimize measurement errors, two trained orthopedic surgeons performed a physical examination of the patients and evaluated the postoperative scars, and the mean values of any measurements were used. The Wilcoxon signed-rank test was used to compare the preoperative and postoperative values in the two study groups. The Mann–Whitney *U* test was used to compare the results between the two patient groups. The improvement in ankle dorsiflexion angle, GDI, GPS, ankle kinematics, ankle kinetics and percent anatomical lengths of gastrocnemius were also compared between groups. Values were presented as median (range). The level of significance was set at *P* < 0.05.

## Results

### Patient Demographic Data and Clinical Outcome

In 27 pediatric patients with cerebral palsy who had undergone endoscopic transverse Vulpius gastrocsoleus recession, there were no complications, such as infections, cast problems or sural nerve injuries. However, one patient underwent a revision open gastrocsoleus recession 1 year 9 months postoperatively, because of a recurred ankle equinus on both sides. During the open surgery, incomplete division of the midline raphe was noted, but there was no scar adhesion because of the difference in incisions between the procedures.

**Figure 3:**
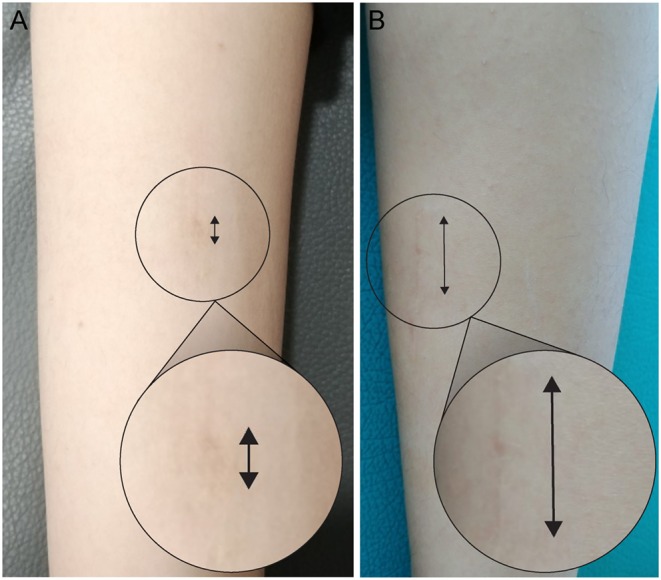
The appearance of scar at final follow-up. The scar was very small and invisible after endoscopic transverse Vulpius gastrocsoleus recession **(A)** compared to open surgery **(B)**.

In the endoscopic transverse Vulpius gastrocsoleus recession surgery group, there were six boys and one girl, with a median age of 7 years (6–10 years). There were three boys and four girls in the open surgical group, with a median age of 8 years (6–10 years). The time for the endoscopic operation was 5 min (range 5–6 min) and that for the open operation was 5 min (range, 4–6 min) (*P* = 0.285) from skin incision to the complete closure. In both groups, there was no significant loss of ankle plantarflexion which demonstrated over-correction or crouch gait at final follow-up. The length of the postoperative scar was smaller in endoscopy group (6.5 mm, range 5–9 mm) compared to open group (28.5 mm, range 20–35 mm) (*P* < 0.001) (Figure 3).

### Comparison of the Physical Examination Between Groups

**Figure 4:**
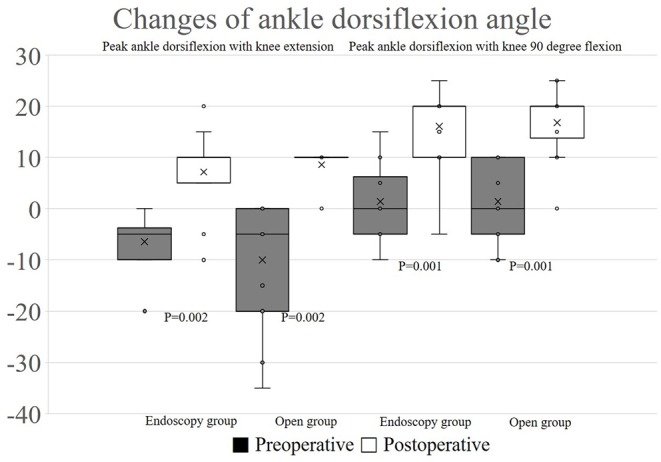
The changes of peak ankle dorsiflexion in each groups.

There were no significant differences in preoperative peak ankle dorsiflexion with knee extension (-5°, range −20 to 0 and −35 to 0, respectively) and peak ankle dorsiflexion with knee 90 degree flexion (0°, range −10 to 15 and −10 to 10, respectively) in both group. Postoperatively, peak ankle dorsiflexion with knee extension (10°, range −10 to 20 and 0 to 10, respectively) and peak ankle dorsiflexion with knee 90 degree flexion (20°, range −5 to 25 and 0 to 25, respectively) demonstrated no statistical difference between groups (Table 1). In both groups, each one leg was recorded as Silfverskiöld positive; however transverse Vulpius gastrocsoleus recession surgery was performed like another leg. Ankle dorsiflexion with knee extension (*P* = 0.002, 0.002, respectively, endoscopy and open group) and ankle dorsiflexion with knee flexion (*P* = 0.001, 0.001, respectively, endoscopy and open group) were increased in both groups after the operations (Figure 4).

**Table 1:** Comparison of clinical evaluation (degrees).

		**Endoscopy group**	**Open group**	***P* value**
Preoperative	Peak ankle dorsiflexion with knee extension	−5 (−20 to 0)	−5 (−35 to 0)	0.946
	Peak ankle dorsiflexion with knee 90 degree flexion	0 (−10 to 15)	0 (−10 to 10)	0.910
Postoperative	Peak ankle dorsiflexion with knee extension	10 (−10 to 20)	10 (0 to 10)	0.427
	Peak ankle dorsiflexion with knee 90 degree flexion	20 (−5 to 25)	20 (0 to 25)	0.910

### Comparison of the Gait Analysis Between Groups

**Figure 5:**
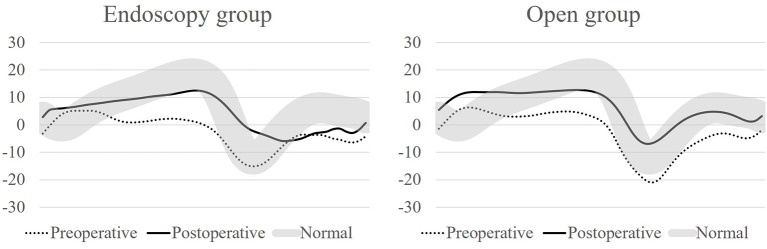
Ankle sagittal kinematics pre- and postoperatively for endoscopy and open group.

There were no significant differences in preoperative gait parameters between the two groups. The maximum ankle dorsiflexion during stance phase was improved from 12.1 degrees (range, −17.2 to 22.7 degrees) to 15.5 degrees (range, 3.4 to 29.6 degrees) in endoscopy group (*P* = 0.011) and from 11.4 degrees (range, −15.2 to 22.7 degrees) to 15.9 degrees (range, 3.2 to 29.1 degrees) in open group (*P* = 0.008) (Figure 5). The gait deviation index (GDI) was improved in endoscopy group (preoperative 54.5, range 26.4–79.1 and postoperative 55.4, range 36.9–98.6, *P* = 0.022) and in open group (preoperative 55.6, range 42.9–73.8 and postoperative 63.1, range 46.2–95.7, *P* = 0.006). The gait profile score (GPS) was also significantly improved in endoscopy group (preoperative 15.3, range 6.7–30.0 and postoperative 13.9, range 6.0–23.3, *P* = 0.022) and in open group (preoperative 14.3, range 9.2–17.8 and postoperative 12.1, range 5.9–17.3, *P* = 0.006).

There was not a significant difference between groups in postoperative gait parameters, except ankle dorsiflexion. The maximum ankle dorsiflexion during stance phase was higher in the open group (15.9°, range 3.4–29.6°) compared with the endoscopy group (15.5°, range 3.2–29.1°) (*P* = 0.009) (Table 2). However, there was not a significant difference in the improvement of maximum ankle dorsiflexion during stance phase between endoscopy (10.9°, range −9.7 to 29.0°) and open (7.4°, range −6.1 to 18.5°) group (*P* = 0.804) and also the improvement of GDI (6.3, range −6.6 to 25.0 in endoscopy group and 6.8, range −6.9 to 21.9 in open group) and GPS (2.4, range −2.6 to 9.9 in endoscopy group and 2.1, range −1.5 to 5.7 in open group) were not differ between groups (*P* = 0.571, 0.454, respectively) (Table S1).

**Table 2:** Comparison of gait deviation index (GDI), gait profile score (GPS), and gait parameters during stance phase.

		**Endoscopy group**	**Open group**	***P* value**
Preoperative	Gait deviation index	54.5 (26.4 to 79.1)	55.6 (42.9 to 73.8)	0.482
	Gait profile score	15.3 (6.7 to 30.0)	14.3 (9.2 to 17.8)	0.329
	Maximum ankle dorsiflexion (degrees)	12.1 (−17.2 to 22.7)	11.4 (−15.2 to 22.7)	0.778
	Maximum ankle plantarflexion moment (Nm/kg)	1.0 (0.8 to 1.4)	0.8 (0 to 1.2)	0.842
	Maximum lateral gastrocnemius percent anatomical length	1.23 (1.12 to 1.31)	1.22 (1.16 to 1.3)	0.606
	Maximum medial gastrocnemius percent anatomical length	1.24 (1.14 to 1.32)	1.23 (1.18 to 1.32)	0.578
Postoperative	Gait deviation index	55.4 (36.9 to 98.6)	63.1 (46.2 to 95.7)	0.194
	Gait profile score	13.9 (6.0 to 23.3)	12.1 (5.9 to 17.3)	0.137
	Maximum ankle dorsiflexion (degrees)	15.5 (3.4 to 29.6)	15.9 (3.2 to 29.1)	0.009
	Maximum ankle plantarflexion moment (Nm/kg)	0.9 (0.6 to 1.1)	1.0 (0.7 to 1.8)	0.936
	Maximum lateral gastrocnemius percent anatomical length	1.22 (1.17 to 1.38)	1.22 (1.18 to 1.38)	0.241
	Maximum medial gastrocnemius percent anatomical length	1.24 (1.17 to 1.34)	1.24 (1.18 to 1.34)	0.596

## Discussion

The gastrocsoleus muscle and tendon unit is divided into three anatomical zones. Zone 2 extends from the medial end of the gastrocnemius muscle, to the end of the soleus muscle and this layer is referred to as the conjoined gastrocnemius aponeurosis-soleus fascia (1, 18). Transverse Vulpius gastrocsoleus recession is a Zone 2 procedure, which lengthens both the gastrocnemius and soleus muscles equally (9, 12, 13). Endoscopic gastrocsoleus recession is an alternative method for the correction of equinus gait and it has shown to result in smaller postoperative scars and protection of the sural nerve due to the improved anatomic visualization with endoscopy (2, 3, 5, 6). However, there was little evidence for the efficacy and safety of endoscopic gastrocsoleus recession in patients with cerebral palsy. We reviewed 27 children with cerebral palsy who had undergone endoscopic transverse Vulpius gastrocsoleus recession and compared the gait improvement in independent ambulatory spastic diplegia between open and endoscopic surgery.

The main advantage of endoscopic transverse Vulpius gastrocsoleus recession surgery is the small incision and in our experience, it was difficult to identify the scar at final follow-up. Previous studies have reported that another advantage of endoscopic surgery was the protection of sural nerve as it can be better identified using the light of endoscopy (2, 4–6). However, we could not identify the sural nerve more easily under endoscopy but we found that there was no need to identify the sural nerve because the skin incision is on the medial side and the procedure is conducted under the fascia. Furthermore, we did not experience any difficulty to protect the sural nerve even during the open surgery.

**Figure 6:**
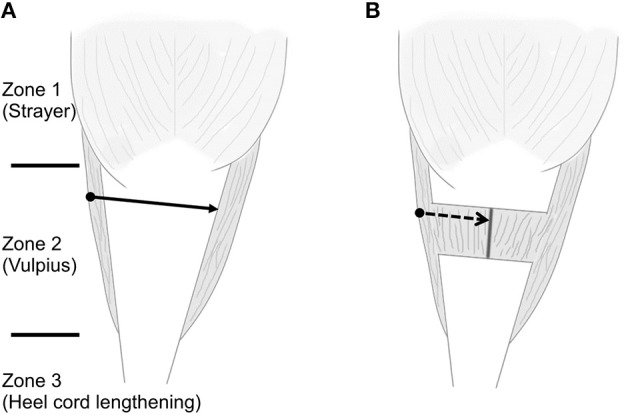
Schematic draw about three zones, different treatment options, and the difficult division of midline raphe. **(A)** The solid arrow is the cutting line of conjoined tendon during endoscopic transverse Vulpius gastrocsoleus recession and the black round circle is the skin incision area. **(B)** After the recession of conjoined tendon, the midline raphé can be seen between the muscle belly of soleus. The direction of endoscopic blade is vertical to the direction of midline raphe (dashed arrow). The deep portion of midline raphe is buried in the muscle belly and it is difficult to cut the deep portion with endoscopic blade.

Although there was no significant complication, one patient who had undergone endoscopy surgery required open revision surgery because of an early recurrence of ankle equinus. The ankle dorsiflexion was improved after endoscopy surgery, but the gait improvement was not so satisfied. At postoperative 1-year, equinus gait has still existed without AFO. During the revision surgery, the soft tissue adhesion was less compared to those after open gastrocsoleus recession, and this may be related to the different incision sites and lack of longitudinal fascia stripping during the endoscopic surgery. We could not identify the exact cause of early recurrence, but a remnant of midline raphé due to incomplete division was noted. We suspect that insufficient muscle lengthening resulted due to the difficulty in the division of the midline raphe (9, 12). The fascia's cutting line in the endoscopic technique is of the coronal plane, however the midline raphé should be cut in the sagittal plane (Figure 6). Therefore, it is difficult to cut the deep portion of midline raphe as it is buried in the muscle belly. If there is insufficient muscle lengthening after the endoscopic surgery, this may result in variable gait improvement.

Endoscopic transverse Vulpius gastrocsoleus recession surgery can be used as part of SEMLS to improve the gait of cerebral palsy patients and as such gait improvement should be a prerequisite comparison treatment outcome. The gait deviation index (GDI) and gait profile score (GPS) were significantly improved in both the open and endoscopic group. Although the improvement was quite small, previous studies have shown that the minimal clinically significant difference for the GPS is a 1.6° difference (19). This may be related to the strict inclusion/exclusion criteria for the comparison of gait improvement to include only independent ambulatory and exclude other bony deformity. The pathologic gait in this study group was only the equinus gait and this is most likely associated with the small improvement of GDI or GPS.

The gait improvement and the increase of ankle dorsiflexion angle during stance phase were noted after both procedures, however the postoperative ankle dorsiflexion angle during stance phase was greater in the open surgery group. Although the lengthening of gastrocsoleus was successful in both techniques, endoscopic transverse Vulpius gastrocsoleus recession was found to be less effective in terms of increasing ankle dorsiflexion angle due to the limitations in releasing the midline raphé. In the physical examination, the median value of preoperative ankle dorsiflexion with the knee in 90-degree flexion was 0°. In a recently published study, the indication for a transverse Vulpius gastrocsoleus recession was suggested to be the presence of a 15° equinus when the knee is in flexion (9). However, we didn't apply this indication previously and so the ankle dorsiflexion in this study was less severe. If the preoperative ankle dorsiflexion was more severe, there might be a difference in the improvement of ankle dorsiflexion and this is a more important clinical outcome for patients with cerebral palsy than a smaller postoperative scar.

In our opinion, when the patient and parent desire a small incision and isolated recession of the gastrocsoleus is required to improve the pathological gait, endoscopic transverse Vulpius gastrocsoleus recession surgery would be considered. However, if the ankle dorsiflexion was not sufficiently achieved, open division of the midline raphé should be considered in children with cerebral palsy. Another disadvantage of the endoscopic procedure may be the additional preoperative preparation time and expense of the endoscopic equipment. Although the operation time from skin incision to closure was about 5 min, the endoscopic technique required more time to prepare. We were unable to compare the additional expense of the endoscopic equipment, because the endoscopic gastrocsoleus recession procedure was not supported by the national health insurance in our country. We did not charge the patients for the endoscopic surgery, so there was no difference in the cost between open and endoscopic group in this study. However, the endoscopy surgery is most likely to be more costly due to the preparation of the equipment. The limitations in the division of the midline raphé, additional time and expense should be considered before the surgical decision is made.

This study was a retrospective study, performed by a single surgeon, and had several limitations. First, the patients used for the comparison of gait improvement were between 6 and 10 years-of-age, with spastic diplegia, bilateral leg operations and GMFCS level II, and due to the strict inclusion criteria, the number of included patients were small. Additionally, a midfoot break may act as a measurement bias because 3-segment foot model had not applied previously. However, there was no differences in the preoperative pathologic gait between two groups because of the strict criteria and they had no bony deformity that may act as a bias. Furthermore, the selection of patients who had undergone this isolated surgery was difficult as it is a component of a SEMLS. There may be a difference in the experience of the surgeon with endoscopic surgery and considering the associated learning curve, we excluded the first 10 patients. In this study, follow-up gait analysis outcomes were compared after 2 years of postoperative follow-up. However, skeletal maturation in growing children with cerebral palsy may affect their gait and long-term follow-up study should be conducted.

In children with cerebral palsy, endoscopic transverse Vulpius recession has the advantage of a minimal and more cosmetic scar. In independent ambulatory patients with spastic diplegia, the significant improvements of ankle dorsiflexion and gait parameters after endoscopic transverse Vulpius recession were confirmed and found to be comparable to the open surgery. However, there may be some difficulties in the division of the midline raphé during the endoscopic procedure, and the insufficient lengthening may relate to undercorrection or result in the early recurrence of ankle equinus. In our opinion, endoscopic transverse Vulpius recession should be recommended for the spastic diplegic patients with mild equinus gait only. Surgeon should be aware the advantages and disadvantages of the endoscopic transverse Vulpius gastrocsoleus recession.

## Data Availability Statement

The datasets generated for this study are available on request to the corresponding author.

## Ethics Statement

The studies involving human participants were reviewed and approved by Haeundae Paik Hospital Institutional Review Board. Written informed consent to participate in this study was provided by the participants' legal guardian/next of kin.

## Author Contributions

D-WK, J-YY, and M-KO: data analysis. HK: supervision. K-BP and D-WK: drafted the manuscript. K-BP and IR: edited and revised the manuscript. All authors have read the manuscript and agreed to its being submitted for publication and individuals listed as authors meet the appropriate authorship criteria, nobody who qualifies for authorship has been omitted from the list. In brief, all authors contributed to the conception and design of the research.

### Conflict of Interest

The authors declare that the research was conducted in the absence of any commercial or financial relationships that could be construed as a potential conflict of interest.
