# Supplementary material for: Endoscopic Transverse Gastrocsoleus Recession in Children With Cerebral Palsy
Source: Front Pediatr. 2020 Mar 24;8:112. doi: 10.3389/fped.2020.00112 (PMC7105772; doi:10.3389/fped.2020.00112)
Supplement: Supplementary file 1 [file Table_1.docx]

**Table S1.** Changes of individual patient’s gait parameters.

| Grooup | ID | Sex | Age | Side | Gait deviation index | | | Gait profile score | | | Maximum ankle dorsiflexion  during stance phase | | |
| --- | --- | --- | --- | --- | --- | --- | --- | --- | --- | --- | --- | --- | --- |
|  |  |  |  |  | Preoperative | Postoperative | Improvement | Preoperative | Postoperative | Improvement | Preoperative | Postoperative | Improvement |
| Endoscopy | 1 | Boy | 7 | Right | 43.09 | 52.84 | 9.75 | 17.53 | 14.25 | 3.28 | -17.15 | 3.6 | 20.75 |
|  |  |  |  | Left | 32.75 | 36.92 | 4.17 | 26.12 | 23.29 | 2.83 | -15.07 | 13.93 | 29.00 |
|  | 2 | Boy | 7 | Right | 73.52 | 98.56 | 25.04 | 9.44 | 6.00 | 3.44 | 15.34 | 22.26 | 6.92 |
|  |  |  |  | Left | 79.12 | 84.18 | 5.06 | 6.67 | 7.82 | -1.15 | 13.52 | 22.6 | 9.08 |
|  | 3 | Boy | 6 | Right | 67.09 | 63.45 | -3.64 | 10.63 | 11.44 | -0.81 | 1.11 | 9.91 | 8.80 |
|  |  |  |  | Left | 52.48 | 63.08 | 10.61 | 15.51 | 11.96 | 3.55 | 13 | 3.35 | -9.65 |
|  | 4 | Boy | 10 | Right | 58.33 | 65.82 | 7.49 | 12.98 | 11.01 | 1.96 | 18.51 | 9.09 | -9.42 |
|  |  |  |  | Left | 60.84 | 54.23 | -6.61 | 12.36 | 14.95 | -2.58 | 18.38 | 29.56 | 11.18 |
|  | 5 | Girl | 7 | Right | 27.35 | 43.56 | 16.21 | 25.36 | 17.79 | 7.58 | 2.22 | 12.96 | 10.74 |
|  |  |  |  | Left | 53.31 | 49.80 | -3.52 | 15.21 | 16.80 | -1.59 | 19.78 | 15.2 | -4.58 |
|  | 6 | Boy | 8 | Right | 57.56 | 56.57 | -0.99 | 13.67 | 13.52 | 0.14 | 12.76 | 24.6 | 11.84 |
|  |  |  |  | Left | 55.65 | 57.82 | 2.17 | 15.38 | 13.52 | 1.86 | 11.19 | 22.23 | 11.04 |
|  | 7 | Boy | 7 | Left | 26.35 | 44.07 | 17.71 | 25.94 | 17.61 | 8.34 | -1.44 | 16.43 | 17.87 |
|  |  |  |  | Right | 28.34 | 42.96 | 14.62 | 30.03 | 20.10 | 9.93 | -9.11 | 15.87 | 24.98 |
| Open | 8 | Girl | 6 | Right | 64.04 | 73.42 | 9.38 | 11.30 | 9.55 | 1.75 | 11.85 | 24.89 | 13.04 |
|  |  |  |  | Left | 54.40 | 62.07 | 7.67 | 15.00 | 12.73 | 2.27 | 22.72 | 29.11 | 6.39 |
|  | 9 | Boy | 9 | Right | 66.64 | 71.52 | 4.88 | 10.48 | 9.35 | 1.13 | 18.6 | 18.52 | -0.08 |
|  |  |  |  | Left | 63.16 | 65.21 | 2.05 | 11.52 | 10.86 | 0.67 | 12.46 | 9.59 | -2.87 |
|  | 10 | Girl | 8 | Right | 55.02 | 51.36 | -3.66 | 13.40 | 14.88 | -1.47 | 14.65 | 20.13 | 5.48 |
|  |  |  |  | Left | 57.22 | 63.13 | 5.91 | 13.64 | 11.69 | 1.95 | 17.32 | 21.34 | 4.02 |
|  | 11 | Girl | 8 | Right | 73.80 | 95.70 | 21.90 | 9.19 | 5.90 | 3.30 | -0.69 | 10.89 | 11.58 |
|  |  |  |  | Left | 67.30 | 72.75 | 5.45 | 10.40 | 8.97 | 1.43 | 10.85 | 4.78 | -6.07 |
|  | 12 | Boy | 7 | Right | 42.92 | 51.19 | 8.27 | 17.78 | 15.24 | 2.54 | -15.22 | 3.23 | 18.45 |
|  |  |  |  | Left | 53.50 | 63.16 | 9.66 | 15.19 | 12.76 | 2.43 | -2.27 | 6.23 | 8.50 |
|  | 13 | Girl | 8 | Right | 49.19 | 46.24 | -2.95 | 15.90 | 17.33 | -1.43 | 4.02 | 13.37 | 9.35 |
|  |  |  |  | Left | 56.11 | 49.25 | -6.85 | 17.12 | 14.37 | 2.75 | 9.2 | 9.63 | 0.43 |
|  | 14 | Boy | 10 | Right | 51.32 | 60.01 | 8.68 | 14.94 | 12.57 | 2.38 | 14.64 | 29.12 | 14.48 |
|  |  |  |  | Left | 49.92 | 65.45 | 15.53 | 16.89 | 11.21 | 5.68 | 5.01 | 23.21 | 18.20 |
